# The Dark Side of Gadolinium: A Study of Arthrographic Contrast at Extreme Concentrations

**DOI:** 10.7759/cureus.6006

**Published:** 2019-10-27

**Authors:** Chandana Kurra, Taylor S Harmon, Kristin Taylor, Joseph Utz, Mauricio Hernandez, Jerry Matteo, Paul Wasserman

**Affiliations:** 1 Radiology, University of Florida College of Medicine, Jacksonville, USA

**Keywords:** hyperconcentrated gadolinium, technical error, simulation of disease, gadolinium, arthrography, magnetic resonance imaging

## Abstract

Magnetic resonance arthrography is used to optimally image the detailed intraarticular anatomy of the joint space. A common iatrogenic complication of arthrography is the extra-articular injection of the gadolinium solution in the periarticular tissues; however, a less common complication involves the abnormal concentration of gadolinium within the prepared injectate. The following describes the inadvertent injection of a hyper-concentrated intraarticular solution of gadolinium and the subsequent appearance that resulted in the post-procedure magnetic resonance imaging examination. In addition, an in-vitro experiment was performed to determine the exact etiology of the abnormal magnetic resonance imaging findings that resulted in this case. The subsequent discussion revisits the signal intensity of gadolinium at extreme concentration ranges and proposes modifications of procedure protocol to mitigate the chance of a repeat event.

## Introduction

Magnetic resonance (MR) arthrography is the modality of choice to investigate the internal derangement of joint spaces. The study includes a preliminary procedural phase that involves the injection of the diluted gadolinium contrast solution into a joint space and a diagnostic phase that involves magnetic resonance imaging (MRI). Given that MR arthrography is a multistep process that involves at least two imaging modalities to successfully deliver the intraarticular contrast and image the patient, there are multiple opportunities for procedural errors to occur. While dilutional errors, in particular, are uncommon, these errors may occur unbeknownst to the physician or technologist until the completion of the MRI stage of the exam [1].

The appearance of abnormally high concentration of intraarticular gadolinium can be mistaken for pathology, resulting in diagnostic errors. Once the error is recognized, reimaging the patient involves rescheduling for a repeat injection, usually several days to weeks later to ensure that all of the contrast has been reabsorbed, returning the joint to its baseline appearance. Given the potential for the patient inconvenience, confusion with billing processes, and most importantly, the uncertain chondrotoxicity of this event, it is important that radiologists familiarize themselves with this type of error and the potential ramifications.

Previous cases of hyper-concentrated gadolinium arthrography have been documented. One such case demonstrated that there was no harm done to a patient who was injected with an aberrant concentration of gadolinium. The resultant MR images of these cases, though, found that the intraarticular space was distorted by profoundly low signal [2]. Another report expressed how the “black” contrast effect of inadvertent administration of excessive gadolinium misrepresents any pre-existing joint pathology and must be followed up with a repeat MR arthrogram after a minimum of five hours [3]. Though most radiologists are aware of the rare possibility for accidental gadolinium overexposure in patients receiving arthrograms, there are limited studies in the literature of the in vivo assessment of MR signal intensity with varying gadolinium concentrations [2].

In 2008, Bleicher and Kanal described the MRI relative signal intensity of two Federal Drug Administration approved gadolinium compounds using serial dilution. For both agents, T1 weighted signal intensities increased, peaked, and subsequently diminished as dilution factors increased and decreased, resembling a bell-shaped pattern. The hypointense or dark signal was seen in the highest and lowest concentrations of the documented serial dilutions, with a subjective optimum signal falling between the two extremes. The report concluded that two commonly used gadolinium contrast agents administered in MR imaging studies have the brightest T1 signal intensities when the serial dilutions are neither too high nor too low in concentration [4].

Though not readily documented, certain in-vivo reports have applied Bleicher and Kanal’s bell-shaped gadolinium curve to analyze optimal enhancement, mainly in the central nervous system [5-8]. Application of the Bleicher and Kanal bell-shaped gadolinium curve has not been applied to MR arthrography, until now. The following case describes a patient that received an inadvertent excess dose of gadolinium during an arthrogram, and the methods used to investigate the etiology of the resulting abnormal imaging findings.

## Materials and methods

A 33-year-old female presented to her orthopedist with chronic left hip pain. A left hip MR arthrogram was ordered for the diagnosis of a suspected labral tear. The patient had no relevant past medical history, and a recent pelvic radiograph performed 29 days prior to the MRI demonstrated no osseous, joint space, or soft tissue abnormalities. Following standard informed consent, history, and physical examination, an MR arthrogram was scheduled.

As per departmental protocol, a technologist prepared and labeled a diluted gadolinium contrast solution using the sterile technique, ultimately combining the components in a 20 ml syringe, which included: 10 ml of Omnipaque™ 240, 10 ml of sterile saline, 0.2 ml of gadolinium, and 0.2 ml of 1/1000 diluted epinephrine.

A fluoroscopically guided arthrogram was performed by an interventional radiologist using 1% lidocaine for local anesthesia, a 22 gauge spinal needle for intraarticular access, and the aforementioned combination of diluted gadolinium solution. Fluoroscopic images revealed contrast distention of the hip joint, confirming a successful intraarticular injection. The patient tolerated the procedure well without any immediate complications. After the contrast solution was injected, the patient obtained an MRI on a 1.2 Tesla open bore magnet (OASIS™, Hitachi Ltd., Tokyo, Japan), according to institutional protocol.

**Figure 1:**
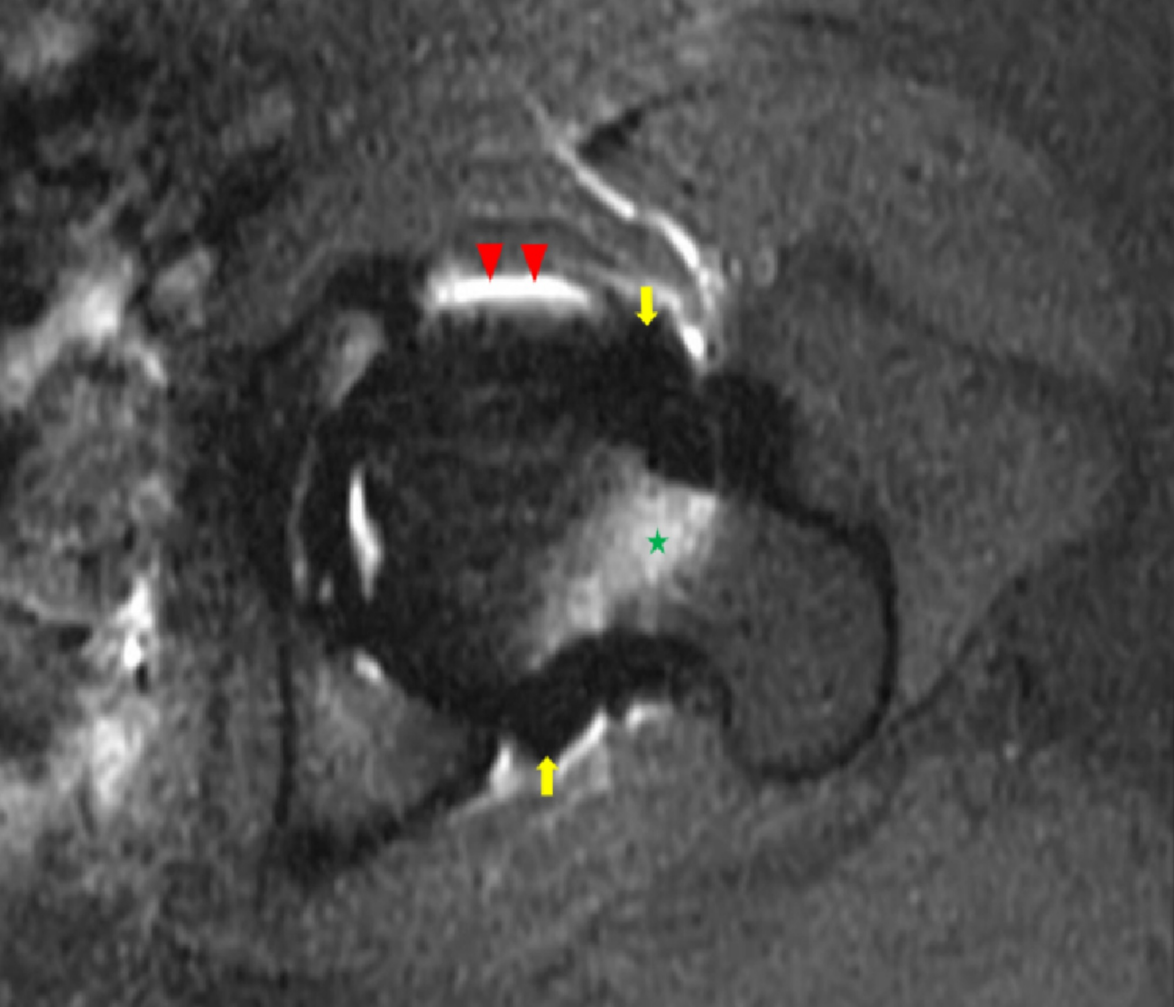
Axial T1 weighted fat-saturated magnetic resonance image of the left hip A T1 weighted axial fat-saturated magnetic resonance image of the left hip exhibits a signal void surrounding the left hip (yellow arrows), conforming to the shape of the joint capsule, status post-injection for magnetic resonance arthrography. There is an incidental note of altered fat saturation at the anterior and posterior joint capsule margins (red arrowheads) and within the marrow of the femoral neck (green star).

**Figure 2:**
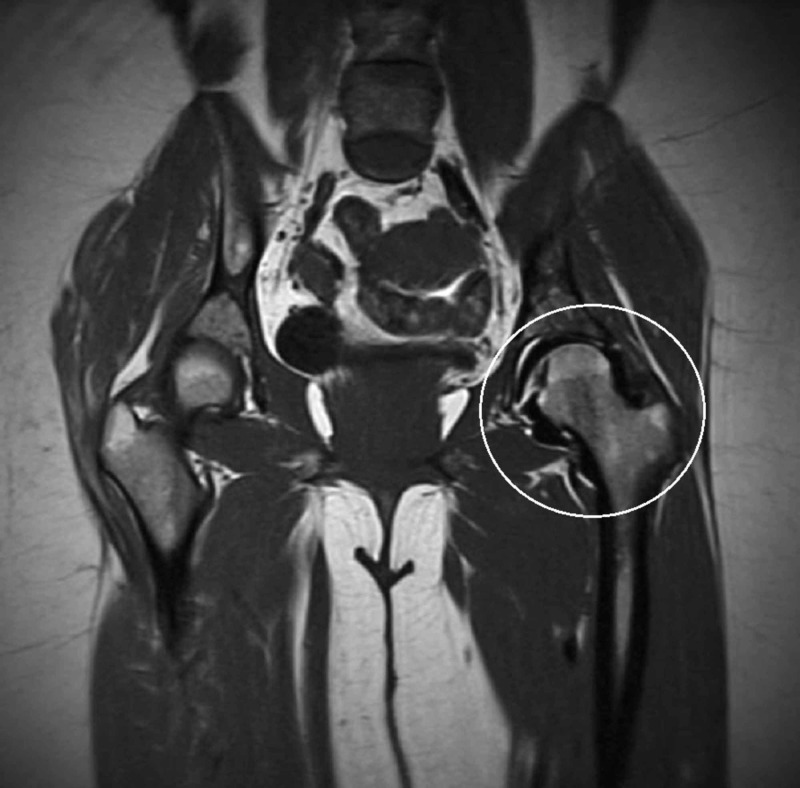
T1 weighted coronal magnetic resonance image of the pelvis The T1 weighted coronal magnetic resonance image of the left hip (white circle) exhibits a signal void surrounding the left hip and conforms to the shape of the joint capsule status post-injection for magnetic resonance arthrography.

An experienced musculoskeletal radiologist’s interpretation of the MRI images revealed homogenously, profoundly hypointense T1 and T2 signal (signal void) abnormality conforming to the boundaries of the left hip joint capsule. No osseous erosions or other abnormalities were identified (Figures 1, 2).

Based on imaging findings and contemplation of potential differential diagnoses, an iatrogenic etiology was proposed involving the inadvertent administration of hyper-concentrated gadolinium into the joint space. After notifying the patient’s ordering physician, it was agreed that a short-term follow-up MRI should be performed to reassess the situation.

**Figure 3:**
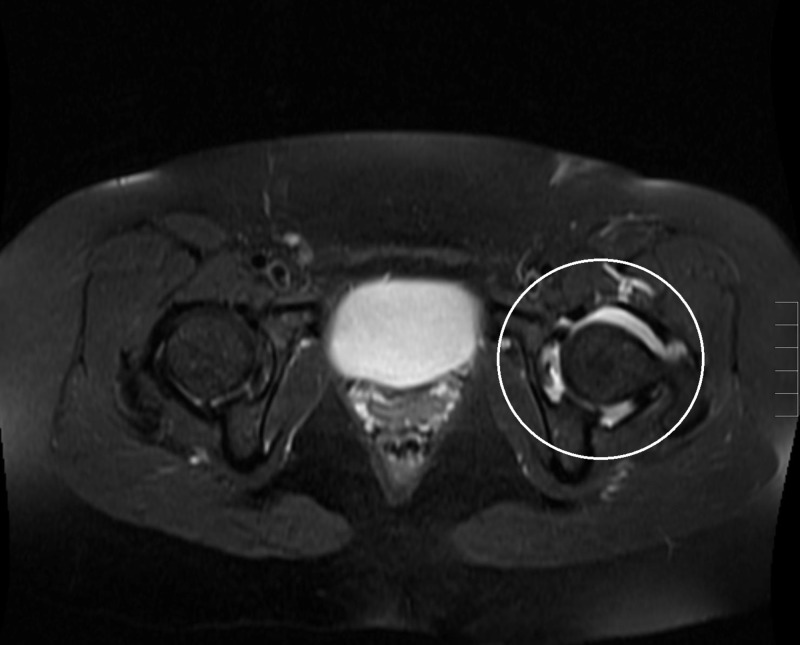
Axial T2 weighted fat-saturated magnetic resonance image of the pelvis A repeated T2 weighted axial fat-saturated magnetic resonance image of the patient's left hip (white circle) revealed the expected appearance, status post intraarticular injection for magnetic resonance arthrography.

**Figure 4:**
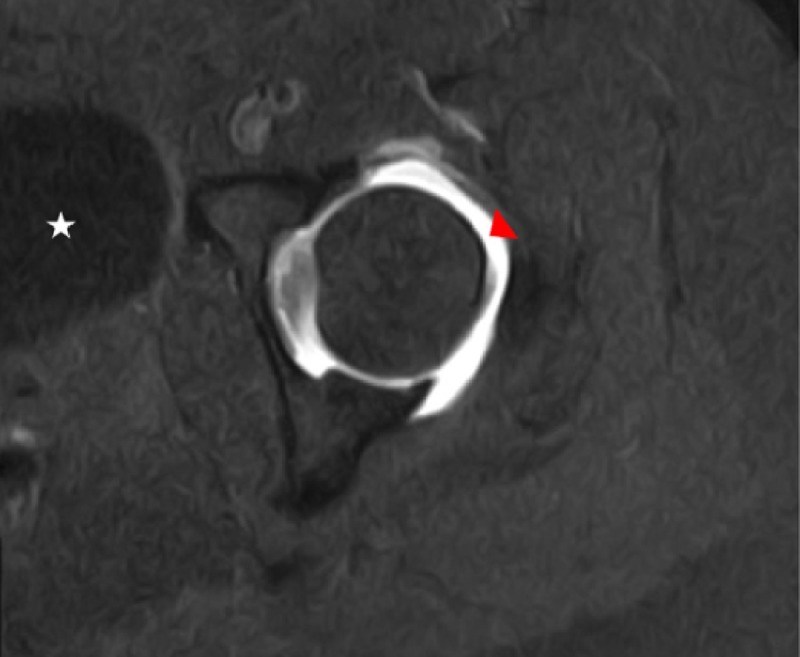
T1 weighted axial fat-saturated magnetic resonance image of the affected left hip A repeated T1 weighted axial fat-saturated magnetic resonance image of the affected left hip status post intraarticular injection for magnetic resonance arthrography is shown. The hyperintense effect of correctly diluted gadolinium (red arrowhead) in comparison to the hypointense appearance of the partially imaged urinary bladder (star) is also demonstrated.

The patient returned two weeks following the original procedure and underwent a repeat MRI, this time without intraarticular contrast utilizing an abbreviated imaging protocol. The MRI revealed the complete resolution of the previously noted diffuse homogeneously low T1 signal associated with the left hip. The appearance of a normal left hip MRI on the abbreviated study ruled out the possibility of intrinsic disease and confirmed an iatrogenic etiology. Following this revelation, during the same day encounter, the patient’s left hip was reinjected with an intraarticular diluted gadolinium solution for the second time. A repeat left hip MR exam revealed a normal joint space with a homogenously bright/hyperintense T1 signal outlining the boundaries of the joint capsule. This study exhibited the normal T1 shortening behavior that is expected for correctly diluted gadolinium-enhanced arthrography (Figures 3, 4).

## Results

**Figure 5:**
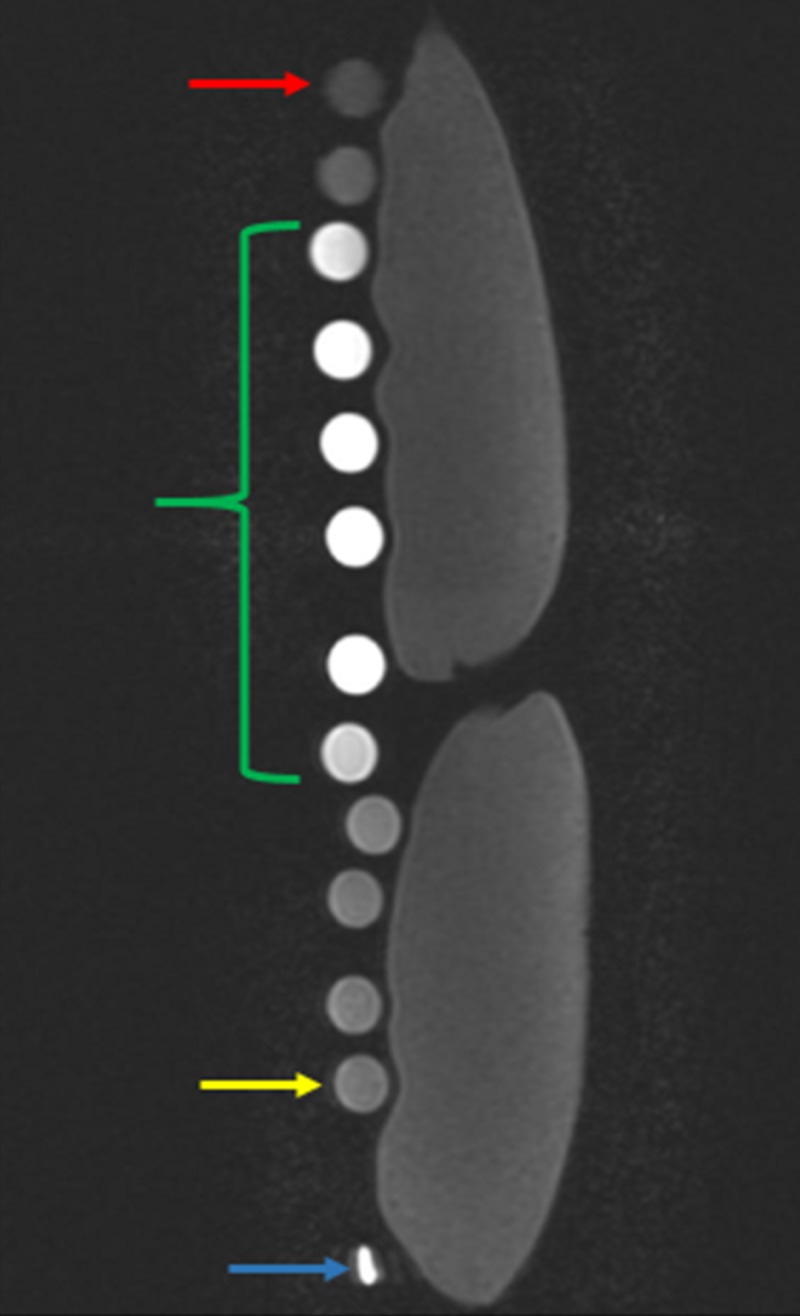
In vitro results of serial diluted gadolinium solution on T1 weighted magnetic resonance imaging The results of the in vitro experiment emulates the Bleicher and Kanal bell-shaped curve, where the test tube with the most and least concentrated gadolinium solutions are represented with the lowest signal intensity at the top (red arrow) and bottom (yellow arrow), respectively. The test tubes with gadolinium solution concentrations suitable for arthrographic joint injections lie within these concentration extremes (green bracket). All test tube samples were imaged adjacent to two 500 milliliter bags of normal saline. The blue arrow represents the magnetic resonance imaging marker.

As an adjunct experiment and to further illustrate the bell-shaped signal intensity of diluted gadolinium as shown in the Bleicher and Kanal study, an in vitro investigation of multiple samples of serially diluted 10 ml vials of gadolinium was performed. The amount of 0.2 ml of gadolinium was used as the mean dilution, corresponding to the standard clinical dilution at the institution the experiment was performed. Using the same T1 parameters as in the clinical protocol, it was confirmed that gadolinium exhibits low signal at extremely high and low concentrations (Figure 5).

**Figure 6:**
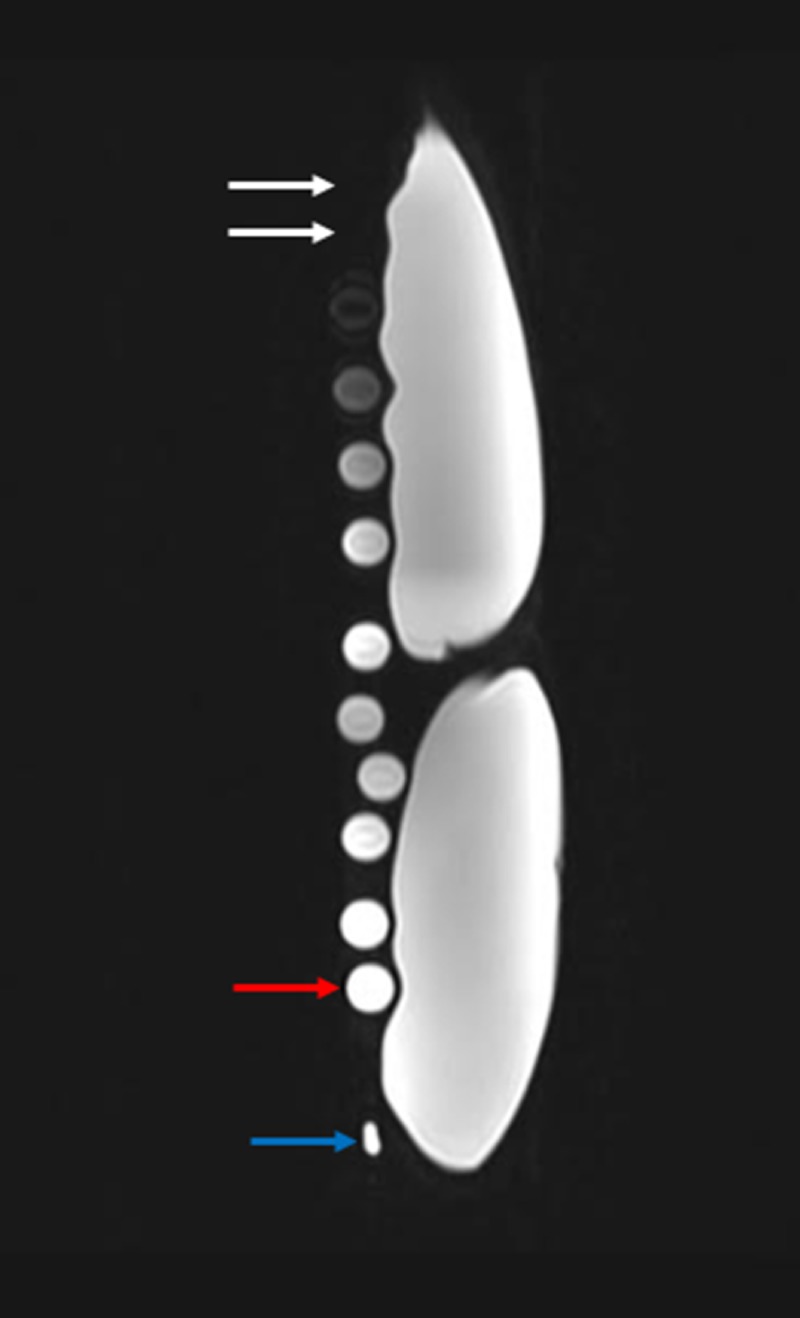
In vitro results of serial diluted gadolinium solution on T2 weighted magnetic resonance imaging The results of the in vitro experiment show the test tube with the most concentrated gadolinium solutions are not visualized, given the low signal intensity (white arrows). The test tube with the most dilute gadolinium solution (red arrow) has the highest signal intensity on T2 weighted magnetic resonance imaging. All test tube samples were imaged adjacent to two 500 milliliter bags of normal saline. The blue arrow represents the magnetic resonance imaging marker.

The tubes were then imaged using the T2 parameters dictated by institutional protocol. The images demonstrate extremely low signal at high gadolinium concentrations, and high signal at low concentrations (Figure 6).

**Figure 7:**
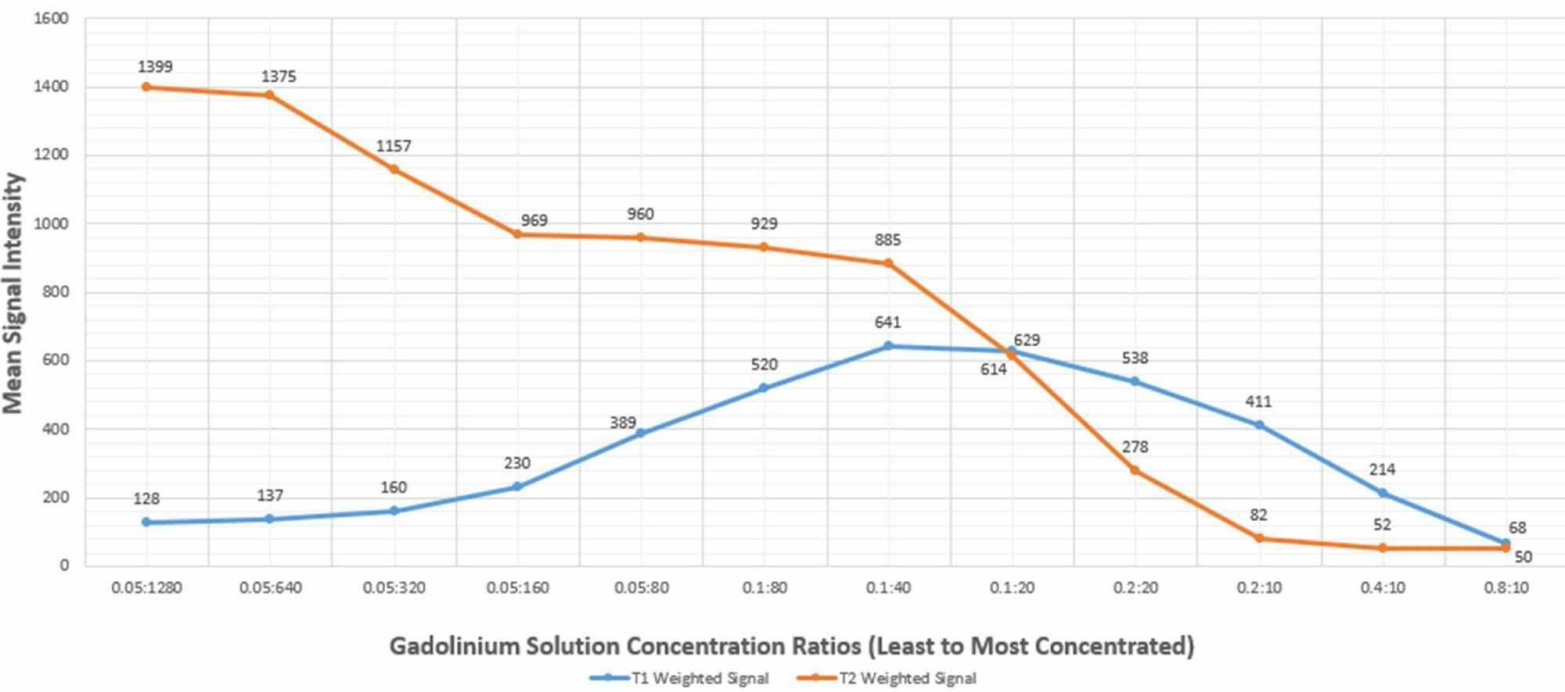
Signal intensity of test tubes at various gadolinium concentrations The figure shows a bell-shaped signal intensity curve of gadolinium on T1 weighted magnetic resonance imaging (blue line) with low signal intensity at both high and low concentrations. The figure also demonstrates the inverse relationship between gadolinium concentrations and signal intensity on T2 weighted magnetic resonance imaging (orange line).

A graph was generated based on the gadolinium dilution concentrations in comparison to their signal intensities measured on Picture Archiving and Communication Systems (PACS) (Figure 7). Each signal intensity measurement was obtained by drawing a 5 mm circular region of interest over the center of the imaged test tubes. The mean signal intensity was recorded based on the values generated by the PACS monitor.

The generated graph depicts curves consistent with the imaging appearance of the test tubes. There are two points at which the curves demonstrate intersecting values; the first intersecting concentration is seen at the 0.1:20 concentration, and the other at the 0.8:10 concentration. At the 0.1:20 concentration, MR arthrographic imaging findings reflect high signal contrast in the joint space on both T1 and T2 weighting. This concentration is considered diagnostic for MR arthrography. one of the industry standards for an arthrography. The second value at which the curves nearly intersect is at the 0.8:10 concentration. It is at this hyper-concentrated level that one would expect dark/signal void of the joint space on T1 and T2 weighted images, as presented in this case.

## Discussion

The preceding study highlights a rarely seen iatrogenic complication of MR arthrography involving the incorrect concentration of gadolinium. The ramification of this error produces altered imaging characteristics that can mislead those who are not aware of this entity. Differential diagnosis of intraarticular air, pigmented villonodular synovitis (PVNS), or hemorrhage, could all be considered if low T1 and low T2 signal material is seen in the joint space. Intraarticular air would produce characteristic "bubbles" of susceptibility artifact, a finding that was not apparent on any of the MR images obtained in this case. When considering PVNS, there is typically a low signal nodular appearance with a “blooming” susceptibility artifact associated with hemosiderin deposition. The abnormality, in this case, had a confluent, non-nodular appearance, without imaging artifacts. Similarly, diffuse hemarthrosis would yield hemosiderin deposition and possibly a hematocrit layering effect. None of these findings were evident in this case, leaving the remaining differential diagnosis of hyper-concentrated intraarticular gadolinium solution as a primary consideration. Furthermore, the normal appearance of the left hip as noted on a short term follow-up MRI strengthens the supposition of an iatrogenic error in the initial visit. It is important to mention that while hypoconcentrated gadolinium exhibits hypointense signal on T1 weighted imaging, and high signal on T2 weighted imaging, hyper-concentrated gadolinium exhibits low signal on both T1 and T2 weighted imaging. The low T2 signal intensity observed at increasing concentrations of gadolinium is the result of decreasing water in solution. Therefore by deduction, it can be concluded that the error encountered in this case was from a hyper-concentrated gadolinium solution.

While the exact cause of this error could not be elucidated in a retrospective analysis, one possible explanation is that instead of adding 10 ml of sterile saline to the solution as per the institutional protocol, 10 ml of gadolinium could have been added inadvertently. The saline and gadolinium are both supplied in 10 ml vials, which could appear similar if viewed at a glance. An alternative, less likely, the explanation could be that the concentration of gadolinium was altered during the manufacturing process.

The preceding case and error prompted a review of arthrographic procedures at the inpatient and outpatient sites of the institution where the patient received care. As a result of the findings, the institution has produced a policy that no longer permits technologists to prepare arthrographic solutions. Moreover, radiologists performing arthrographic procedures now must prepare the gadolinium solution shortly before injection. While this policy does not completely eliminate the risk of dilution errors, it does comply with United States Pharmacopeia 797 recommendation regarding non-physician compounding of pharmaceuticals [9]. Other institutions have enlisted their pharmacies to prepare all arthrographic solutions, and subsequently, deliver the preparation to the radiology department shortly before the injection time [10]. Using the pharmacy in this role removes all compounding responsibility from the radiology department.

While the patient in concern did not have any contemporary ill-effects, and the overall systemic dose of gadolinium was less than seen in most MR studies requiring intravenous gadolinium contrast enhancement, this case does raise the question of chondrotoxicity. Long-term effects on the cartilage of patients exposed to increased concentrations of intraarticular gadolinium have not been thoroughly studied; however, there is a threshold concentration that is known to be chondrotoxic to cartilage in vitro [11].

## Conclusions

Radiologists should be aware of the bell-shaped signal intensity curve that gadolinium demonstrates on T1 imaging in order to recognize the potential complication of hyperconcentrated intraarticular gadolinium. Furthermore, even though it may be an uncommon error, radiologists should be able to recognize the differences between an inadvertent hyperconcentrated gadolinium injection and other intraarticular pathology. Finally, physicians should be mindful of the possibility of a dilutional error during the preparation of gadolinium solutions for MR arthrography.
